# A Common Variant of *NGEF* Is Associated with Abdominal Visceral Fat in Korean Men

**DOI:** 10.1371/journal.pone.0137564

**Published:** 2015-09-04

**Authors:** Hyun-Jin Kim, Jin-Ho Park, Seungbok Lee, Ho-Young Son, Jinha Hwang, Jeesoo Chae, Jae Moon Yun, Hyuktae Kwon, Jong-Il Kim, Belong Cho

**Affiliations:** 1 Institute of Health and Environment, School of Public Health, Seoul National University, Seoul, South Korea; 2 Department of Family Medicine, Seoul National University Hospital, Seoul, South Korea; 3 Genomic Medicine Institute (GMI), Medical Research Center, Seoul National University, Seoul, South Korea; 4 Department of Biochemistry, Seoul National University College of Medicine, Seoul, South Korea; 5 Department of Biomedical Sciences, Seoul National University Graduate School, Seoul, South Korea; 6 Department of Family Medicine, Healthcare Research Institute, Seoul National University Hospital Healthcare System Gangnam Center, Seoul, South Korea; University of Catanzaro Magna Graecia, ITALY

## Abstract

Central adiposity, rather than body mass index (BMI), is a key pathophysiological feature of the development of obesity-related diseases. Although genetic studies by anthropometric measures such as waist circumference have been widely conducted, genetic studies for abdominal fat deposition measured by computed tomography (CT) have been rarely performed. A total of 1,243 participants who were recruited from two health check-up centers were included in this study. We selected four and three single-nucleotide polymorphisms (SNPs) in *NGEF* and *RGS6*, respectively, and analyzed the associations between the seven SNPs and central adiposity measured by CT using an additive, dominant, or recessive model. The participants were generally healthy middle-aged men (50.7 ± 5.3 years). In the additive model, the rs11678490 A allele of *NGEF* was significantly associated with total adipose tissue, visceral adipose tissue (VAT), and subcutaneous adipose tissue (all *P* < 0.05). The AA genotype of this SNP in the recessive model showed a more significant association with all adiposity traits, and its association with VAT remained significant even after adjustment for BMI (*P* = 0.005). In the overall or visceral obesity group analysis, the AA genotype of rs11678490 showed no association with overall obesity (*P* = 0.148), whereas it was significantly associated with visceral obesity both before (*P* = 0.010) and after (*P* = 0.029) adjustment for BMI. In particular, an AA genotype effect was conspicuous between lower and upper groups with 5% extreme VAT phenotypes (OR = 9.59, 95% CI = 1.50–61.31). However, we found no significant association between SNPs of *RGS6* and central adiposity. We identified a visceral-fat-associated SNP, rs11678490 of *NGEF*, in Korean men. This study suggests that the genetic background of central adiposity and BMI is different, and that additional efforts should be made to find the unique genetic architecture of intra-abdominal fat accumulation.

## Introduction

Obesity, which is defined as abnormal or excessive fat accumulation, is a major risk factor for the development of hypertension, type 2 diabetes mellitus, dyslipidemia, cardiovascular diseases, and cancers [1–5]. These obesity-related diseases are mediated by different regional fat distributions, such as visceral and subcutaneous adiposity. To date, many studies have shown that visceral and subcutaneous adiposity play different roles in health outcomes. Visceral adiposity has been demonstrated to be causally related to obesity-related metabolic and cardiovascular diseases [6–9], whereas subcutaneous adiposity might have protective effects in these disorders [10–11]. Although waist circumference (WC) is an alternative easy tool that can be used for the measurement of general central obesity in clinical practice, it is also unable to discriminate this regional fat distribution. Quantitative measurement of central adiposity by computed tomography (CT) has been found to be the most reliable and useful method for regional fat assessment and research on obesity-related complications [12,13].

Numerous family and twin studies have suggested that obesity is strongly influenced by genetic factors [14]. Therefore, many recent large-scale genome-wide association studies (GWASs) were aimed at identifying obesity-related genes. However, most of these studies focused on BMI-associated genes and found only a few common variants with small effect sizes [15–17]. When considering that central adiposity, as represented by visceral and subcutaneous adiposity, is the pathological core phenomenon of obesity-related complications, and that each obesity type might have different effects on health, genetic studies based on accurate and direct measurement of adipose tissue compartments by CT are required for understanding the genetic architecture of obesity. Unfortunately, few GWASs of central adiposity, such as visceral and subcutaneous fat, have been conducted. In particular, GWASs of adipose tissue depots in Asian populations have not been reported. In 2009, Norris et al. first carried out a GWAS and a follow-up analysis of CT-derived phenotypes in Hispanics, and found that two candidate genes, *RGS6* and *NGEF*, influenced the body fat distribution and amount of fat, respectively [18]. In 2012, one European ancestry-based GWAS revealed that a novel single-nucleotide polymorphism (SNP), rs1659258, was associated with visceral adipose tissue (VAT) in women via sex-specific analyses of body fat distribution [19].

Many genetic studies have failed to replicate the loci identified, for several reasons, such as discrepancy in minor allele frequency (MAF) and differences in genetic architecture among populations, thereby emphasizing the necessity of independent replication in other populations. The genetic study of adiposity traits in Asian populations in particular is essential for understanding the genetic background of fat distribution in Asians, because Asians are more likely to have high rates of visceral fat deposition compared with other populations [12,20,21].

This study was aimed at investigating the genetic effects of *NGEF* and *RGS6* on central adiposity traits measured by CT in Korean adult men. We assessed the association between candidate genes and adiposity traits, and provide the first report of the association between central adiposity and SNPs in an Asian population.

## Materials and Methods

### Ethics statement

We obtained written informed consent from all study participants, and this study was approved by the institutional review board of the Seoul National University Hospital Biomedical Research Institute (*approval number*, *H-0911-010-299*).

### Subjects

Participants were recruited from two health check-up centers (the Seoul National University Hospital Health Promotion Center and the Seoul National University Hospital Healthcare System Gangnam Center). A total of 1,399 subjects who visited our centers for periodic comprehensive health check-ups and expressed interest in the study from December 2009 to November 2011 were screened by a family physician. In the subsequent detailed interview, 1,243 subjects who met the inclusion criteria (i.e., (1) subjects who did not take any medications that may affect body weight, such as corticosteroids, antidiabetics, thyroid drugs, and weight-reduction drugs; (2) subjects who underwent an obesity-related procedure or surgery; (3) subjects without necessary phenotypic information; and (4) subjects with a qualified DNA sample) were included in the final analysis (Table 1 and S1 Fig).

**Table 1:** Characteristics of Study Subjects.

	Site A of recruitment (n = 777)		Site B of recruitment (n = 466)		Total (n = 1,243)		
Characteristics	Mean	SD	Mean	SD	Mean	SD	*P*-value^a^
Age (years)	50.7	5.3	49.9	5.3	50.4	5.3	0.022
Weight (kg)	70.9	9.5	72.2	8.7	71.4	9.2	0.014
BMI (kg/m^2^)	24.4	2.7	24.7	2.6	24.5	2.7	0.122
TAT (cm^2^)	261.5	93.0	277.7	89.1	267.5	91.9	0.003
VAT (cm^2^)	125.5	51.8	139.7	50.7	130.8	51.8	<0.001
SAT (cm^2^)	136.0	52.2	137.9	49.0	136.7	51.0	0.530
VSR	0.95	0.3	1.05	0.3	0.99	0.3	<0.001

Abbreviations: BMI, body mass index; TAT, total adipose tissue; VAT, visceral adipose tissue; SAT, subcutaneous adipose tissue; SD, standard deviation

^a^The calculated *P*-value was obtained by independent samples t-test (two-side *P*-value).

### Obesity assessment

Anthropometric measurements were performed on participants in an overnight fasting state who were wearing light clothing. BMI was calculated as weight in kilograms divided by the square of the height in meters (kg/m^2^). Central adiposity, including total, visceral, and subcutaneous adiposity, was measured by abdominal CT scanning (Somatom Sensation 16 CT scanner, Siemens AG, Erlangen, Germany) using a single-slice image taken at the umbilicus level measuring 5 mm in thickness, and the cross-sectional surface areas of the abdominal fat compartments were calculated using the Rapidia 2.8 CT software (Infinitt, Seoul, Korea) within a range of -250 to -50 Hounsfield units, as described previously [22]. The VAT and subcutaneous adipose tissue (SAT) boundaries were defined with a manual tracing method by a cursor. The VAT area was defined by delineating intra-abdominal fat bound by parietal peritoneum or tarnsversalis fascia, excluding vertebra and spinal muscles. SAT area was defined as fat tissue located between inside of dermis and outside of back and abdominal muscle. Total adipose tissue (TAT) is calculated as the sum of VAT and SAT. In addition, we calculated the visceral-to-subcutaneous ratio (VSR).

### SNP genotyping

The procedure used for selecting SNPs is described in S2 Fig. Based on the study reported by Norris et al., first, we selected eight and 12 SNPs for *NGEF* and *RGS6*, respectively. Among them, SNPs with a MAF < 0.05 in HapMap phase III Asian data (JPT and CHB) were excluded. Next, we considered the minimum number of SNPs per candidate gene to avoid redundant SNPs within a gene region for an appropriate multiple-comparison correction. Thus, we assessed linkage disequilibrium (LD) relations between SNPs using HapMap phase III Asian data (JPT and CHB), and only the SNPs which were in low LD relationship (pairwise r^2^ < 0.5) were included for this study. In addition, if the LD level between SNP pairs was moderate or high (pairwise r^2^ ≥ 0.5), only SNPs with the lowest p-values in Norris’s study were selected. Finally, a total of four and three SNPs in *NGEF* and *RGS6*, respectively, were included in this study. We extracted genomic DNA from whole-blood samples using the QuickGene DNA whole-blood kit with QuickGene-610L equipment (Fujifilm, Tokyo, Japan), according to the manufacturer’s standard protocols. All subjects were genotyped by TaqMan SNP Genotyping Assay (Applied Biosystems, Inc., Carlsbad, California, USA); the number of successfully genotyped samples per SNP is indicated in Table 2.

**Table 2:** The list of selected seven SNPs.

population of reference	Gene (Chr)	SNP	Position^a^	Type	Allele	Minor allele (MAF)	N^b^	Genotype count	HWE *P*-value
Hispanic Americans	*NGEF* (2q37)	rs11678490	233830950	Intron	A, G	A (0.32)	1,222	129/529/564	0.793
		rs6745724	233854241	Intron	A, G	A (0.07)	1,228	8/147/1073	0.243
		rs884089	233866029	Intron	G, C	C (0.37)	1,182	165/549/468	0.852
		rs1104953	233870366	Intron	G, C	G (0.16)	1,167	32/303/832	0.507
	*RGS6* (14q24)	rs847354	72781929	Intron	A, G	A (0.32)	1,156	116/514/526	0.591
		rs7147236	72788246	Intron	A, G	G (0.17)	1,227	31/363/833	0.274
		rs6574069	72839100	Intron	C, T	C (0.50)	1,188	277/624/287	0.092

Abbreviations: Chr, chromosome; SNP, single nucleotide polymorphism; MAF, minor allele frequency; HWE, Hardy-Weinberg equilibrium

^a^SNP positions are based on Genome Reference Consortium Human Build 37 (GRCh37).

^b^N is the number of adult samples that were finally genotyped in this study.

### Statistical analysis

To meet the test’s normality assumptions, we checked the distribution of our adiposity traits before analyses. Because all traits followed a non-normal distribution, the best transformation approaches for normality were considered. First, we used natural log and square root transformation methods; however, the transformed traits were still not normally distributed (Shapiro–Wilk *P* < 0.05). Therefore, we applied a rank-based inverse normal transformation to our traits, which is robust to deviations from normality and to outliers. To compare the distribution of each trait between two sample-recruitment sites, we used an independent samples t-test. The Hardy–Weinberg equilibrium (HWE) test for each SNP was performed using PLINK software (version 1.9) [23], and all seven SNP met a criterion for HWE (all *P* > 0.05) (Table 2). We carried out multiple linear regression analyses to identify associations between quantitative adiposity traits and three different genetic models: additive, dominant, and recessive models. These results were adjusted for age and site of recruitment. To assess statistical significance, we considered both the nominal threshold value (*P* < 0.05) and Bonferroni correction for multiple testing (*P* < 0.05/7 = 0.007). A logistic regression analysis of rs11678490 of *NGEF* in binary groups with overall obesity (BMI ≥ 25 kg/m^2^) and visceral obesity (VAT ≥ 136 cm^2^) by transforming quantitative continuous traits to binary traits in total subjects (n = 1,243) was also performed using the three different genetic models. In addition, we used an exact logistic regression model for small sample size for the extreme groups of VAT phenotype, 5% (n = 62) and 10% (n = 124). For all statistical analyses, SAS software (version 9.3) was used.

## Results

### Demographic and anthropometric characteristics

The demographic and anthropometric characteristics of the study subjects who met all inclusion criteria are summarized in Table 1 according to recruitment center and as the combined total group. A total of 1,243 subjects from site A (n = 777) and site B (n = 466) were included in the final analysis. The participants were predominantly middle-aged men (50.4 ± 5.3 years), and subject age in site A (50.7 ± 5.3 years) was somewhat higher than that in site B (49.9 ± 5.3 years) (*P* = 0.022). The BMI distribution was similar between the two recruitment sites (*P* for BMI = 0.122), and the mean value of BMI in the total sample was 24.5. The mean values of SAT were not significantly different between sites A (136.0 ± 52.2) and B (137.9 ± 49.0) (*P* = 0.530), whereas the mean value of VAT in site B (139.7 ± 50.7) was much higher than that in site A (125.5 ± 51.8) (*P* < 0.001).

### Candidate SNPs and adiposity-related traits

The genetic characteristics of the seven candidate SNPs are shown in Table 2. The expected genotype frequencies of all seven candidate SNPs were in Hardy–Weinberg equilibrium (all *P* > 0.05). A multiple linear regression analysis after adjustment for recruitment site and age was performed to assess the genetic association between adiposity-related traits and the seven SNPs (Tables 3 and 4). Among the four intronic SNPs in *NGEF*, only rs11678490’s A allele was significantly associated with TAT, VAT, and SAT except for VSR in the additive genetic model (all *P* < 0.05) (Table 3). In the recessive model, rs11678490’s AA genotype was significantly associated with all central adiposity traits (all *P* < 0.05), and passed the conservative Bonferroni significance threshold of 0.007 (= 0.05/7), which corrected for multiple comparison testing (all *P* < 0.007). We also evaluated associations with VAT after adjusting for BMI, to check whether *NGEF* is a visceral-fat-specific gene after controlling for the degree of overall adiposity. As a result, the significant effect of rs11678490 for VAT in the additive model disappeared (*P* = 0.084), whereas the association signal observed for VAT in the recessive model remained significant (*P* = 0.005). We also checked the association between this SNP and SAT after adjusting for BMI, but found no significant associations in any of the genetic models (data not shown). Similarly, we investigated the genetic effects of the three SNPs located in the intronic region of *RGS6* on adiposity-related traits (Table 4). None of the SNPs showed significant effects on those traits.

**Table 3:** Associations of *NGEF* gene variants with obesity-related index: multivariate linear regression analysis.

		BMI		TAT		VAT		SAT		VSR		BMI-adj VAT	
SNP	Genetic model	*β* (SE)	*P-*value	*β* (SE)	*P-*value	*β* (SE)	*P-*value	*β* (SE)	*P-v*alue	*β* (SE)	*P-* value	*β* (SE)	*P*-value
rs11678490	additive	0.06 (0.04)	0.152	**0.09 (0.04)**	**0.035**	**0.09 (0.04)**	**0.034**	**0.09 (0.04)**	**0.047**	0.02 (0.04)	0.640	0.05 (0.03)	0.084
	dominant	0.03 (0.06)	0.639	0.03 (0.06)	0.579	0.03 (0.06)	0.570	0.05 (0.06)	0.372	-0.01 (0.06)	0.879	0.02 (0.04)	0.576
	recessive	**0.22 (0.09)**	**0.020**	**0.34 (0.09)**	**0.0003**	**0.34 (0.09)**	**0.0002**	**0.26 (0.09)**	**0.005**	0.11 (0.09)	0.209	**0.19 (0.07)**	**0.005**
rs6745724	additive	0.03 (0.08)	0.697	0.06 (0.08)	0.436	0.03 (0.08)	0.735	0.07 (0.08)	0.394	-0.07 (0.08)	0.359	0.01 (0.06)	0.886
	dominant	0.04 (0.09)	0.650	0.07 (0.09)	0.385	0.04 (0.08)	0.664	0.08 (0.09)	0.343	-0.07 (0.08)	0.375	0.01 (0.06)	0.844
	recessive	-0.05 (0.35)	0.888	-0.04 (0.35)	0.904	-0.10 (0.35)	0.772	-0.05 (0.35)	0.900	-0.14 (0.35)	0.678	-0.04 (0.26)	0.864
rs884089	additive	0.02 (0.04)	0.647	-0.00 (0.04)	0.920	-0.02 (0.04)	0.620	0.02 (0.04)	0.699	-0.03 (0.04)	0.467	-0.03 (0.03)	0.288
	dominant	0.04 (0.06)	0.552	-0.00 (0.06)	0.951	-0.04 (0.06)	0.534	0.03 (0.06)	0.655	-0.05 (0.06)	0.369	-0.06 (0.04)	0.154
	recessive	0.01 (0.08)	0.948	-0.01 (0.08)	0.910	-0.01 (0.08)	0.919	0.01 (0.08)	0.894	-0.01 (0.08)	0.865	-0.01 (0.06)	0.927
rs1104953	additive	0.03 (0.06)	0.628	0.03 (0.06)	0.601	0.02 (0.06)	0.775	0.03 (0.06)	0.560	-0.04 (0.05)	0.465	0.00 (0.04)	0.971
	dominant	0.02 (0.06)	0.699	0.04 (0.06)	0.515	0.02 (0.06)	0.716	0.05 (0.06)	0.456	-0.05 (0.06)	0.458	0.01 (0.05)	0.808
	recessive	0.08 (0.18)	0.636	-0.02 (0.18)	0.891	-0.02 (0.18)	0.921	-0.04 (0.18)	0.838	-0.05 (0.18)	0.787	-0.07 (0.13)	0.579

Abbreviations: BMI, body mass index; TAT, total adipose tissue; VAT, visceral adipose tissue; SAT, subcutaneous adipose tissue; VSR, visceral-to-subcutaneous ratio; SNP, single nucleotide polymorphism; SE, standard error

These results were adjusted for age and site of recruitment.

The tested allele is minor allele of each SNP.

Nominally significant results are indicated in bold (*P* < 0.05).

**Table 4:** Associations of *RGS6* gene variants with obesity-related index: multivariate linear regression analysis.

		BMI		TAT		VAT		SAT		VSR		BMI-adj VAT	
SNP	Genetic model	*β* (SE)	*P-*value	*β* (SE)	*P-*value	*β* (SE)	*P-*value	*β* (SE)	*P-*value	*β* (SE)	*P-*value	*β* (SE)	*P-*value
rs847354	additive	0.05 (0.04)	0.242	0.05 (0.04)	0.273	0.05 (0.04)	0.279	0.03 (0.04)	0.530	0.03 (0.04)	0.518	0.01 (0.03)	0.660
	dominant	0.05 (0.06)	0.427	0.04 (0.06)	0.494	0.04 (0.06)	0.466	0.02 (0.06)	0.798	0.03 (0.06)	0.592	0.01 (0.04)	0.769
	recessive	0.12 (0.10)	0.217	0.12 (0.10)	0.208	0.11 (0.10)	0.249	0.09 (0.10)	0.344	0.05 (0.10)	0.601	0.03 (0.07)	0.636
rs7147236	additive	-0.07 (0.05)	0.188	-0.05 (0.05)	0.375	-0.03 (0.05)	0.528	-0.05 (0.05)	0.320	0.00 (0.05)	0.922	0.01 (0.04)	0.729
	dominant	-0.08 (0.06)	0.182	-0.08 (0.06)	0.216	-0.05 (0.06)	0.388	-0.09 (0.06)	0.148	0.01 (0.06)	0.840	0.00 (0.04)	0.989
	recessive	-0.08 (0.18)	0.659	0.13 (0.18)	0.483	0.08 (0.18)	0.653	0.18 (0.18)	0.334	-0.05 (0.18)	0.786	0.15 (0.13)	0.263
rs6574069	additive	-0.01 (0.04)	0.818	0.01 (0.04)	0.822	0.01 (0.04)	0.875	0.02 (0.04)	0.653	0.01 (0.04)	0.843	0.02 (0.03)	0.536
	dominant	-0.03 (0.07)	0.707	0.01 (0.07)	0.878	0.00 (0.07)	0.961	0.02 (0.07)	0.718	0.01 (0.07)	0.912	0.03 (0.05)	0.568
	recessive	0.00 (0.07)	0.996	0.01 (0.07)	0.832	0.01 (0.07)	0.837	0.03 (0.07)	0.714	0.01 (0.07)	0.833	0.02 (0.05)	0.667

Abbreviations: BMI, body mass index; TAT, total adipose tissue; VAT, visceral adipose tissue; SAT, subcutaneous adipose tissue; VSR, visceral-to-subcutaneous ratio; SNP, single nucleotide polymorphism; SE, standard error

These results were adjusted for age and site of recruitment.

The tested allele is minor allele of each SNP.

### Associations of rs11678490 in the visceral obesity group

In Table 3, we demonstrate the contribution of rs11678490’s A allele (*NGEF*) to visceral fat mass, especially in the recessive genetic model. We also assessed the association between rs11678490 and the ‘general’ obesity group (case n = 489, control n = 754), which was defined as subjects with a BMI ≥ 25 kg/m^2^, and the ‘visceral’ obesity group (case n = 559, control n = 684), which was defined as subjects with VAT ≥ 136 cm^2^, which is an optimal cut-off criterion in Korean men (Table 5) [24]. We found no significant association between the overall obesity group and the rs11678490 SNP. However, in the recessive model, rs11678490’s AA genotype was associated with an increased risk of visceral obesity (odds ratio (OR) = 1.63, 95% CI = 1.13–2.37; *P* = 0.010). After adjusting for BMI, rs11678490 retained a statistically significant effect on the visceral obesity group (OR = 1.59, 95% CI = 1.05–2.40; *P* = 0.029). Consequently, in the visceral obesity group, we reconfirmed that *NGEF* contributes to abdominal visceral fat, independently of BMI.

**Table 5:** rs11678490 variant and its association with both overall and visceral adiposity group: multivariate logistic regression analysis.

			Overall obesity group (case n = 489, control n = 754)			visceral adiposity group (case n = 559, control n = 684)					
			BMI ≥ 25kg/m^2^			VAT ≥ 136cm^2^ ^a^			BMI group-adjVAT ≥ 136cm^2^ ^a^		
Gene	SNP	Genetic model	*β* (SE)	OR (95% CI)	*P*-value	*β* (SE)	OR (95% CI)	*P*-value	*β* (SE)	OR (95% CI)	*P*-value
*NGEF*	rs11678490	additive	0.09 (0.09)	1.10 (0.92–1.30)	0.307	0.14 (0.09)	1.15 (0.97–1.36)	0.117	0.12 (0.10)	1.13 (0.93–1.37)	0.212
		dominant	0.05 (0.12)	1.06 (0.84–1.33)	0.641	0.06 (0.12)	1.06 (0.84–1.33)	0.629	0.04 (0.13)	1.04 (0.81–1.34)	0.753
		recessive	0.27 (0.19)	1.31 (0.91–1.90)	0.148	**0.49 (0.19)**	**1.63 (1.13–2.37)**	**0.010**	**0.46 (0.21)**	**1.59 (1.05–2.40)**	**0.029**

Abbreviations: BMI, body mass index; VAT, visceral adipose tissue; SNP, single nucleotide polymorphism; SE, standard error; OR, odds ratio; CI, confidence interval

These results were adjusted for age and site of recruitment.

The tested allele is minor allele of each SNP.

Significant results are indicated in bold (*P* < 0.05).

^a^This value is optimal cut-off in Korean men.

### rs11678490 variant and extreme visceral adiposity

We also performed a case–control association study of the rs11678490 variant and extreme visceral adiposity group defined as subjects within upper and lower 5% visceral adiposity area (n = 62) and 10% (total n = 124) of total sample, respectively (Table 6). The mean VAT of the lower and upper groups in the 10% extreme group were 34.2 ± 11.2 and 247.4 ± 31.1, respectively, and the mean values of each group in the 5% group were 24.5 ± 6.3 and 267.4 ± 33.9, respectively (data not shown). Compared to the allele frequency (AF) of total sample (AF = 0.32), the risk AFs of rs11678490 (A allele) increased in both 5% (AF = 0.45) and 10% extreme upper groups (AF = 0.37) (data not shown). In the 10% extreme group, the risk of visceral obesity in subjects with the AA genotype increased 3-fold compared with those with the AG or GG genotype (OR = 3.33, 95% CI = 1.12–9.90; *P* = 0.031). This genetic association remained significant after adjusting for BMI (OR = 7.79, 95% CI = 1.50–40.47; *P* = 0.015). In addition, we investigated the effect of the association in the 5% extreme group among the total sample. Compared with the 10% extreme group, the effect size of the AA genotype in the 5% extreme group was larger (OR = 9.59, 95% CI = 1.50–61.31; *P* = 0.017).

**Table 6:** Recessive model results of rs11678490 for 5% and 10% extreme group of visceral adiposity distribution.

	Count n (%)		logistic results of VAT extreme group			BMI group-adj logistic results of VAT extreme group		
The cut-off % of Extreme	The lower VAT group	The upper VAT group	*β* (SE)	OR (95% CI)	*P*-value	*β* (SE)	OR (95% CI)	*P*-value
**5% (Total n = 62)**								
AG or GG	30 (96.8)	21 (67.7)	**2.26 (0.95)**	**9.59 (1.50–61.31)**	**0.017**	**4.30 (1.90)**	**73.46 (1.76 –>999.99)**	**0.024**
AA	1 (3.2)	10 (32.3)						
Total	31 (100.0)	31 (100.0)						
**10% (Total n = 124)**								
AG or GG	57 (91.9)	48 (77.4)	**1.20 (0.56)**	**3.33 (1.12–9.90)**	**0.031**	**2.05 (0.84)**	**7.79 (1.50–40.47)**	**0.015**
AA	5 (8.1)	14 (22.6)						
Total	62 (100.0)	62 (100.0)						

Abbreviations: VAT, visceral adipose tissue; BMI, body mass index; SE, standard error; OR, odds ratio; CI, confidence interval

These exact logistic results were adjusted for age and site of recruitment.

The tested allele is minor allele of each SNP.

Significant results are indicated in bold (*P* < 0.05).

## Discussion

This study was performed to assess the genetic effects of *NGEF* and *RGS6* on central adiposity traits, including visceral fat, in an Asian population. We measured TAT, VAT, and SAT area using CT, as well as BMI, in Korean adult men, and assessed the genetic associations between *NGEF* and *RGS6* and central adiposity traits using four and three SNPs in the *NGEF* and *RGS6* genes, respectively. We found that the A allele of the intronic SNP rs11678490 of *NGEF* was associated with TAT, VAT, and SAT in the additive model (all *P* < 0.05) and with all adiposity-related traits in the recessive model (all *P* < 0.05). After adjusting for BMI, the genetic effect of this SNP regarding VAT in the recessive model remained significant, suggesting the possibility of an abdominal visceral-fat-specific gene. Interestingly, that effect was conspicuous between lower and upper groups with 5% extreme VAT phenotypes (OR = 9.59, 95% CI = 1.50–61.31). In contrast, we found no significant associations between central adiposity traits and SNPs in *RGS6*.

Reports of visceral fat distribution vary according to ethnicity and sex. Asian populations generally have greater VAT levels than do other populations under conditions such as the same age and WC, despite the presence of substantially lower levels of overall obesity [12,20,21]. In addition, the abdominal VAT level is much higher in men than in women because women tend to store fat in the hips or thighs as opposed to abdominal in men [19]. In the study performed by Norris et al., which was based on Hispanic Americans, the mean VAT of the entire cohort was 114.7 [18], whereas the mean value of VAT in this study, which included only men, was 130.8. We showed a higher prevalence of intra-abdominal obesity; however, the mean age of subjects in Norris et al.’s study (mean age = 42.8) has a little younger than in our study (mean age = 50.4), and the proportion of women was over 50%. For this reason, this inconsistency regarding visceral fat deposition between the two studies is thought to be the result of age, sex proportion, and ethnic differences.

We identified a significant genetic association between VAT and rs11678490 of *NGEF*, which plays a critical role in the formation of neuronal connections. In the additive genetic model, our results for *NGEF* were consistent with those of Norris et al., raising the possibility of the existence of a total fat-contributing gene. However, in the recessive genetic model, the effect of the SNP on VAT after controlling for BMI remained (*P* < 0.007). This result suggests a new hypothesis: that *NGEF* contributes to abdominal visceral fat, as well as to overall adiposity. Interestingly, this possibility was also identified in further association analysis of the visceral obesity group, or groups with 5% and 10% extreme values of VAT distribution. In particular, the effect size of rs11678490, despite the fact that this is a common variant, increased remarkably in the group with extreme values of VAT phenotype. Compared with the results of Norris et al., our distinctive results for *NGEF* can be explained by several possible factors. First, the assumed genetic model in each study was different. The associations of this SNP in Norris et al.’s study were tested by an additive genetic model [18], whereas we used all possible genetic models, i.e., additive, dominant, and recessive models. As a result, we found a stronger genetic effect of rs11678490 in the recessive model. Second, this may be because of a discrepancy in MAF between the two populations. The MAF of rs11678490 in our study (MAF = 0.32) was greater than that reported in Hispanic Americans (MAF = 0.19) [18]. Therefore, this variant may be an Asian-specific SNP that influences visceral fat deposition, independently of overall obesity estimated by BMI. This SNP may explain, to some extent, the greater VAT levels observed in Asian populations. In addition, this may indicate a sex-specific effect of rs11678490 on VAT.

The present study had several new aspects. First, we provided a significant replication result of *NGEF* for adiposity phenotypes using CT data. Most genetic studies of overall or abdominal obesity have used nonspecific anthropometric traits, such as BMI and WC, rather than CT measures, which are associated with a high cost. In this sense, our more precise and refined traits for body fat distribution, including VAT and SAT, may enable the significant replication of the effect of *NGEF*, despite the relatively smaller sample size compared with those of large-scale GWASs. Moreover, we performed comprehensive genetic analyses of visceral fat for the first time in an Asian population, the results of which suggest the new possibility that a variant of *NGEF* in the recessive genetic model contributes to the distribution of adiposity, particularly VAT. However, we were unable to identify these associations in women, as only men were included in this study. In 2012, one association study reported a sex-specific genetic effect on visceral fat [19]. They performed a GWAS of the distribution of fat among people with European ancestry, and found that a novel variant, rs1659258, which is located on chromosome 2, was related to visceral fat in women, but not in men. This may be because of the known sex differences in the distribution of abdominal fat. To determine the sex-specific effect of our *NGEF* SNP on VAT, further genetic association studies in women are needed.

In conclusion, we aimed to investigate the genetic effects of central adiposity with 7 SNPs of two candidate genes in a Korean population. We identified that VAT is associated with SNP rs11678490 of *NGEF* in Korean men.

## Supporting Information

S1 Fig The selection of study subjects for this study. (DOCX)

S2 Fig The Selection of SNPs for *NGEF* and *RGS6* gene. (DOCX)
