# Supplementary figures and images for: A Common Variant of NGEF Is Associated with Abdominal Visceral Fat in Korean Men
Source: PLoS One. 2015 Sep 4;10(9):e0137564. doi: 10.1371/journal.pone.0137564 (PMC4560439; doi:10.1371/journal.pone.0137564)

**S1 Fig. The selection of study subjects for this study.**


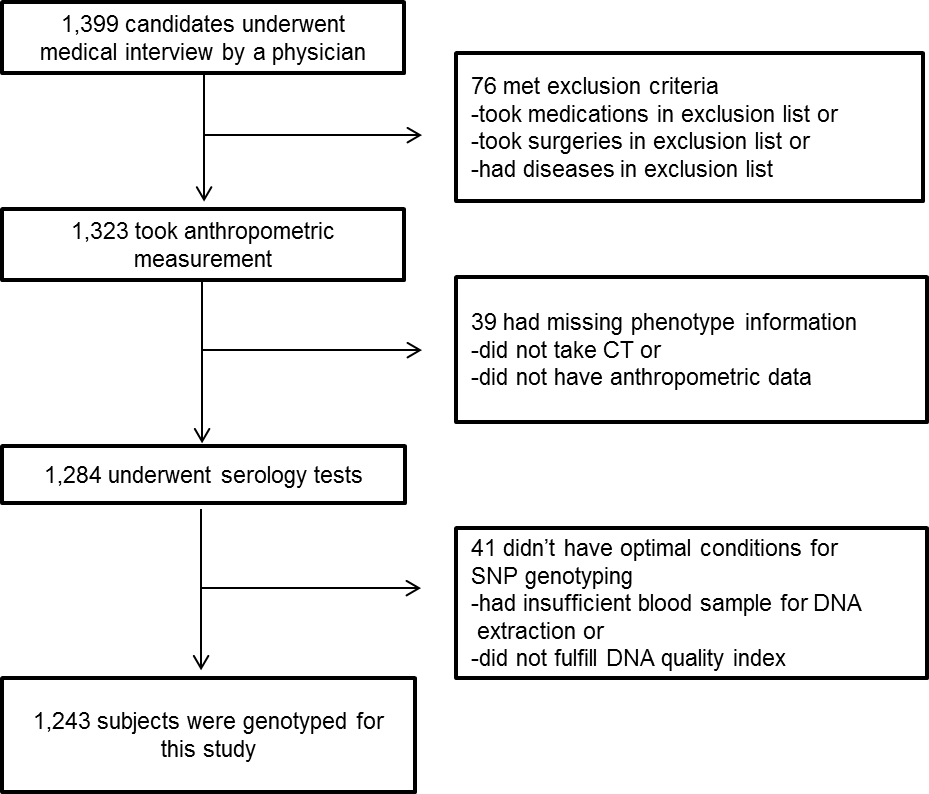

Supplement: S1 Fig — (DOCX) [file pone.0137564.s001.docx]

**S2 Fig. The Selection of SNPs for *NGEF* and *RGS6* gene.**

**
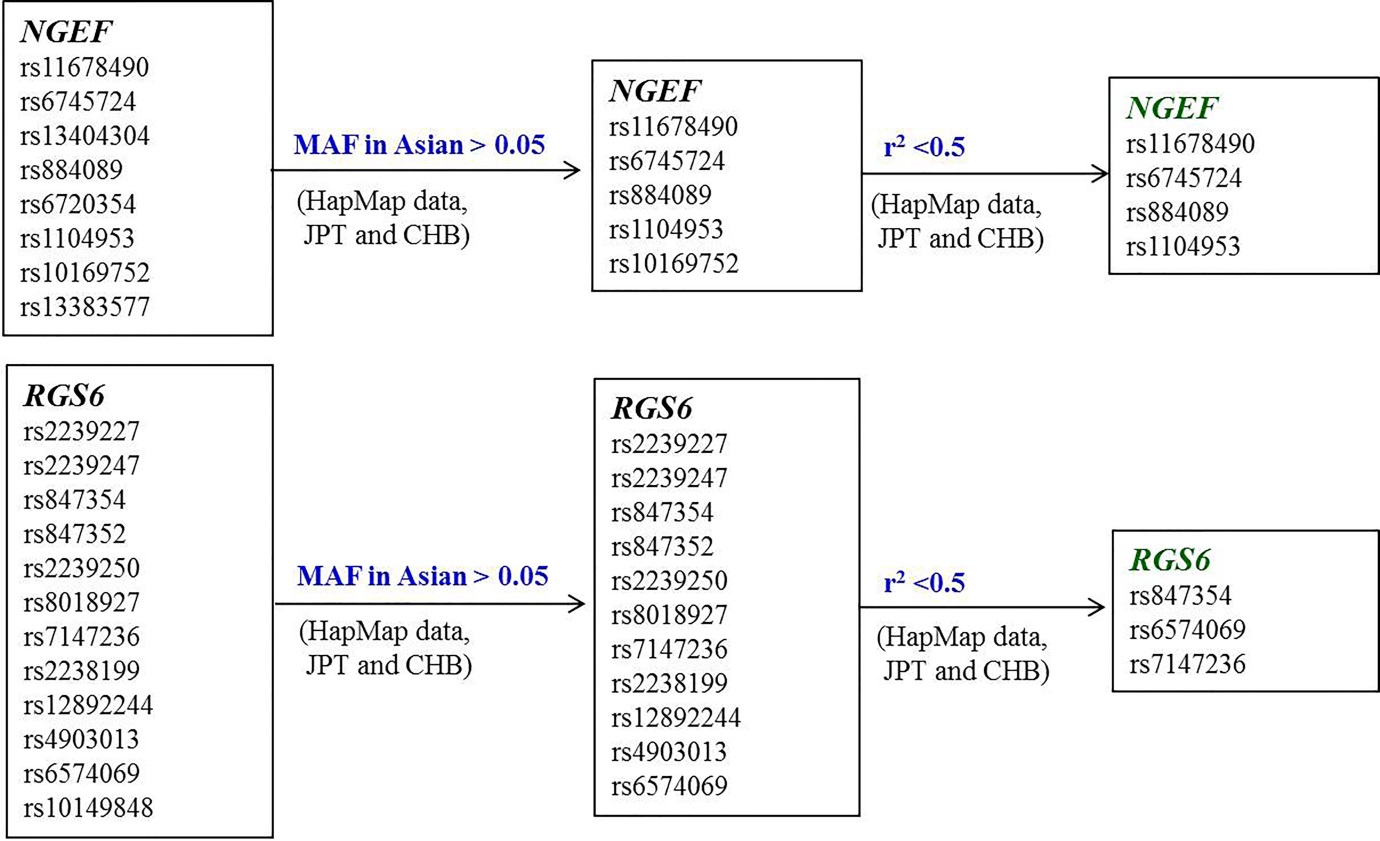
**

Supplement: S2 Fig — (DOCX) [file pone.0137564.s002.docx]
